# The Comparative Effectiveness of Ceftolozane/Tazobactam versus Aminoglycoside- or Polymyxin-Based Regimens in Multi-Drug-Resistant *Pseudomonas aeruginosa* Infections

**DOI:** 10.3390/antibiotics11050626

**Published:** 2022-05-06

**Authors:** Aisling R. Caffrey, Haley J. Appaneal, J. Xin Liao, Emily C. Piehl, Vrishali Lopes, Ryan J. Dillon, Laura A. Puzniak, Kerry L. LaPlante

**Affiliations:** 1 Infectious Diseases Research Program, Providence Veterans Affairs Medical Center, Providence, RI 02908, USA; haley.appaneal@gmail.com (H.J.A.); jxliao@uri.edu (J.X.L.); ec.piehl@gmail.com (E.C.P.); vrishali.lopes@va.gov (V.L.); kerrylaplante@uri.edu (K.L.L.); 2 Center of Innovation in Long-Term Support Services, Providence Veterans Affairs Medical Center, Providence, RI 02908, USA; 3 College of Pharmacy, University of Rhode Island, Kingston, RI 02881, USA; 4 School of Public Health, Brown University, Providence, RI 02903, USA; 5 Merck & Co., Inc., Rahway, NJ 07065, USA; ryan.dillon@merck.com (R.J.D.); laurapuzniak@yahoo.com (L.A.P.); 6 Warren Alpert Medical School, Division of Infectious Diseases, Brown University, Providence, RI 02903, USA

**Keywords:** aminoglycosides, ceftolozane/tazobactam, comparative effectiveness, multi-drug resistant, polymyxins, *Pseudomonas aeruginosa*

## Abstract

*Pseudomonas aeruginosa* infections are challenging to treat due to multi-drug resistance (MDR) and the complexity of the patients affected by these serious infections. As new antibiotic therapies come on the market, limited data exist about the effectiveness of such treatments in clinical practice. In this comparative effectiveness study of ceftolozane/tazobactam versus aminoglycoside- or polymyxin-based therapies among hospitalized patients with positive MDR *P. aeruginosa* cultures, we identified 57 patients treated with ceftolozane/tazobactam compared with 155 patients treated with aminoglycoside- or polymyxin-based regimens. Patients treated with ceftolozane/tazobactam were younger (mean age 67.5 vs. 71.1, *p* = 0.03) and had a higher comorbidity burden prior to hospitalization (median Charlson 5 vs. 3, *p* = 0.01) as well as higher rates of spinal cord injury (38.6% vs. 21.9%, *p* = 0.02) and *P. aeruginosa*-positive bone/joint cultures (12.3% vs. 0.7%, *p* < 0.0001). Inpatient mortality was significantly lower in the ceftolozane/tazobactam group compared with aminoglycosides or polymyxins (15.8% vs. 27.7%, adjusted odds ratio 0.39, 95% confidence interval 0.16–0.93). There were no significant differences observed for the other outcomes assessed. In hospitalized patients with MDR *P. aeruginosa*, inpatient mortality was 61% lower among patients treated with ceftolozane/tazobactam compared to those treated with aminoglycoside- or polymyxin-based regimens.

## 1. Introduction

*Pseudomonas aeruginosa* infections are challenging to treat due to multi-drug resistance (MDR) and the complexity of the patients affected by these serious infections [1]. As new antibiotic therapies come on the market, limited data exist on the effectiveness of such treatments in clinical practice among varied clinical populations [2]. Aminoglycosides and polymyxins have been used to treat MDR *P. aeruginosa* infections but present significant toxicity profiles, including nephrotoxicity, ototoxicity, and neurotoxicity [3,4,5]. Additionally, these agents are associated with suboptimal pharmacokinetics, narrow therapeutic index, and inferior efficacy [4,5,6,7]. Historically, aminoglycosides and polymyxins have been used to treat resistant *P. aeruginosa* infections. Still, clinical data defining optimal dosing and combination regimens of these agents are lacking [2,8].

*P. aeruginosa* resistance to aminoglycosides and polymyxins generally remains low (<4% of MDR isolates). Therefore, the benefit–risk profile of these antibiotics pushes the scale toward benefits outweighing risks in MDR and extensively resistant infections [9]. Alternatively, novel anti-pseudomonal antibiotics may be preferred if they result in at least similar rates of positive clinical outcomes without the safety concerns. To date, there have only been select comparative effectiveness studies comparing ceftolozane/tazobactam-based regimens, a novel cephalosporin/beta-lactamase inhibitor combination, with aminoglycoside or polymyxin-based regimens, in patients with MDR *P. aeruginosa* infections [7,10]. Just one study in the United States has been conducted, including 100 ceftolozane/tazobactam treated patients and 100 aminoglycoside or polymyxin-treated patients from 6 hospitals in Michigan and Ohio [7]. This study found that acute kidney injury was significantly lower in the ceftolozane/tazobactam group (adjusted odds ratio [aOR] 0.08; 95% confidence interval [CI], 0.03–0.22), while no difference was observed in inpatient mortality (aOR 0.62, 95% CI 0.30–1.28). We, therefore, conducted a comparative effectiveness study of ceftolozane/tazobactam versus aminoglycosides or polymyxins in another clinical population, patients treated in Veterans Affairs (VA) hospitals nationally, to assess the real-world benefit–risk profile of ceftolozane/tazobactam compared with aminoglycosides or polymyxins for the treatment of MDR *P. aeruginosa* infections. 

## 2. Results

**Figure 1:**
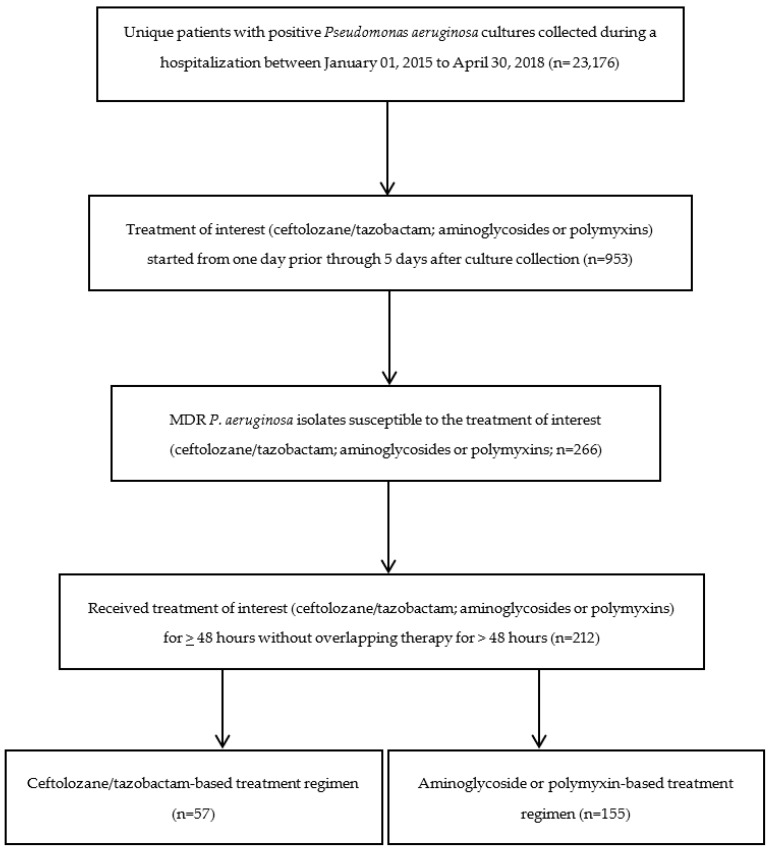
Flow chart for the study of cohort identification.

We identified 23,176 hospitalized patients with positive *P. aeruginosa* cultures between January 2015 and April 2018 from 62 medical centers. After applying the inclusion/exclusion criteria shown in (Figure 1), we identified 212 patients with a MDR *P. aeruginosa* infection, of which 26.9% (n = 57) were treated with ceftolozane/tazobactam, and 73.1% (n = 155) were treated with aminoglycoside- or polymyxin-based regimens. Of the 155 patients in the aminoglycoside or polymyxin treatment group, 132 received aminoglycoside-based therapy (48 tobramycin, 50 amikacin, and 34 gentamicin) and 23 received polymyxin-based therapy (0 colistin, 23 polymyxin B).

Baseline demographics and clinical characteristics are presented in Table 1. Patients treated with ceftolozane/tazobactam-based regimens were younger (mean age 67.5 vs. 71.1, *p* = 0.03) and less likely to be of Hispanic or Latino ethnicity (1.8% vs. 22.6%, *p* < 0.001) than patients treated with aminoglycoside- or polymyxin-based regimens. Those treated with ceftolozane/tazobactam also had a higher comorbidity burden (median Charlson 5 vs. 3, *p* = 0.01) as well as higher rates of spinal cord injury (38.6% vs. 21.9%, *p* = 0.02) and positive *P. aeruginosa* cultures in the 30 days before admission (54.4% vs. 38.7%, *p* = 0.04) than those treated with aminoglycoside- or polymyxin-based regimens. Patients treated with ceftolozane/tazobactam were more likely to be admitted from a nursing home (12.3% vs. 3.2%, *p* = 0.02) and to have had a previous nursing home stay (5.3% vs. 0%, *p* = 0.02). There were also significant differences in the *P. aeruginosa* culture source. A bone/joint source of MDR *P. aeruginosa* was more common in those treated with ceftolozane/tazobactam (12.3% vs. 0.7%, *p* < 0.001), and a urine source was less common (22.8% vs. 51.6%, *p* < 0.001).

Concomitant treatments were similar between treatment groups, except for meropenem treatment (Table 2). Patients treated with ceftolozane/tazobactam-based regimens were less likely to be treated with concomitant meropenem (15.8% vs. 30.3%, *p* = 0.03) than those treated with aminoglycoside- or polymyxin-based regimens. Patients treated with ceftolozane/tazobactam were more likely to be treated with meropenem (24.6% vs. 11.6%, *p* = 0.02) and piperacillin/tazobactam (35.1% vs. 16.8%, *p* = 0.004) in the 30 days before the treatment of interest than those treated with aminoglycoside- or polymyxin-based regimens. The median time to initiation of the treatment of interest from MDR *P. aeruginosa* culture collection was three days for both groups.

The overall inpatient mortality rate was 24.5% (52/212). After controlling for confounders, inpatient mortality was significantly lower in patients treated with ceftolozane/tazobactam-based regimens than with aminoglycoside or polymyxin-based regimens (15.8% vs. 27.7%, aOR 0.39, 95% CI 0.16–0.93; Table 3). No significant differences were observed for any of the other clinical outcomes assessed, including acute kidney injury (16.7% vs. 12.4%, aOR 0.86, 95% CI 0.32–2.33).

## 3. Discussion

In hospitalized patients with MDR *P. aeruginosa* infections, we found a significantly lower risk of in-hospital mortality among patients treated with ceftolozane/tazobactam-based regimens compared with those treated with aminoglycoside- or polymyxin-based regimens in the national VA Healthcare system. We did not observe differences in 30-day readmission, persistent positive culture, microbiological clearance, or acute kidney injury.

Our national study population had important similarities and differences from the previous comparative effectiveness study conducted in the United States [7]. Our study population was older (mean age 70 years), more male (99%), and more white (69%) than previous work (mean age 59 years, 68% male, and 58% white). In both studies, most patients presented from home (37% of our study vs. 39% previous work), and chronic pulmonary disease, diabetes, and congestive heart failure were common comorbidities. However, the median Charlson Comorbidity Index was higher in our study (4) than in previous work (3) [7]. Most patients in the previous study were admitted to the ICU (69%), and the most common infection type was ventilator-associated pneumonia (52%), with 46% of patients with sepsis. A total of 59% of our patients received intensive care during admission, with only 3.8% and 24% of our patients being diagnosed with pneumonia and sepsis, respectively. Based on culture data, only 36% of our patients had a pseudomonal respiratory tract infection. Patients with pneumonia generally have worse outcomes, and pneumonia is an independent risk factor for death in patients with pseudomonal infections [11,12]. Therefore, patients in previous work may have been more severely ill, leading to differences in the study findings. Interestingly, however, inpatient mortality rates were similar in both studies (25% of our study vs. 23% in previous work) [7]. 

Additionally, there was higher concomitant antibiotic therapy in both treatment groups in our study compared to previous work, with 96% in the ceftolozane/tazobactam and 94% in the aminoglycoside- or polymyxin-based treatment group having received concomitant antibiotic therapy in our study, as compared to 15% in the ceftolozane/tazobactam and 72% in the aminoglycoside or polymyxin treatment group in previous work [7]. Importantly, we assessed differences between the treatment groups in concomitant therapy from the index date (initiation of ceftolozane/tazobactam or aminoglycosides/polymyxins) through 15 days after the index date, while previous work only considered combination therapy with secondary agents also targeting *P. aeruginosa* for >48 h. Moreover, unlike our study, the previous study did not consider differences in treatments leading up to the index date, including previous antibiotics for past infections and empiric therapy for the treatment of the MDR *P. aeruginosa* infection. These important differences in treatment may have impacted the therapy chosen (ceftolozane/tazobactam vs. aminoglycoside or polymyxin-based regimens) and clinical outcomes.

We found no difference in clinical outcomes assessed except inpatient mortality. In previous work, receipt of ceftolozane/tazobactam was associated with increased clinical cure (aOR 2.63; 95% confidence interval [CI], 1.31–5.30) and decreased acute kidney injury (AKI, aOR, 0.08; 95% CI, 0.03–0.22) [7]. We did not assess clinical cure. Clinical cure is a subjective clinical outcome defined as improvement in symptoms. However, improvement in symptoms can occur from administering non-antibiotic medications (e.g., fever reducing, anti-inflammatory, steroids). Further, clinical cure may be defined differently between clinicians and investigators [7,13]. Another small, matched case-control study which included 48 patients from 9 medical centers in Italy with nosocomial pneumonia or bloodstream infection due to MDR or extensively drug-resistant *P. aeruginosa* found no difference in clinical cure (13% vs. 18%, *p* = 0.11) and decreased acute kidney injury (0 vs. 8%, *p* = 0.04) among those treated with ceftolozane/tazobactam versus colistin- or aminoglycoside-based regimens [10]. We did not find any difference in acute kidney injury in our study (aOR, 0.86; 95% CI, 0.32–2.33).

In both the American comparative effectiveness study and the Italian case-control study, similar survival rates were observed among those treated with ceftolozane/tazobactam versus aminoglycoside- or polymyxin-based regimens [7,10]. However, the previous study of patients in the United States may have detected a difference had they had a larger sample size (at least 250 per group) assuming a crude incidence of in-hospital mortality of 20% vs. 25% and an aOR of 0.62. Previous work among patients in Italian medical centers was also likely underpowered to detect a survival benefit [10]. We observed a benefit in in-hospital survival with ceftolozane/tazobactam. Consistent with our findings, a single-center case-control study among 57 patients with hematologic malignancy and *P. aeruginosa* infection, 50.9% due to MDR and 29.8% to extensively drug-resistant strains, demonstrated a survival benefit associated with ceftolozane/tazobactam [14]. In this single-center case-control study, the 30-day mortality rate was lower among patients treated with ceftolozane/tazobactam versus standard of care antibiotics (5.3% vs. 28.9%; *p* = 0.045) [14].

Among those with known MDR *P. aeruginosa* infections, our results and previous work suggest that treatment with ceftolozane/tazobactam may better meet antimicrobial stewardship goals to optimize outcomes and minimize unintended consequences of aminoglycoside- or polymyxin-based treatment, when the isolates are ceftolozane/tazobactam-susceptible [15]. Our results build on previous findings that in patients with serious infections due to MDR *P. aeruginosa*, ceftolozane/tazobactam has been associated with higher rates of clinical cure and lower rates of nephrotoxicity than aminoglycoside- or polymyxin-based treatment, and ceftolozane/tazobactam may also be associated with an inpatient survival benefit [7,10]. As such, our results support previous studies which have concluded that ceftolozane/tazobactam may be preferred over aminoglycoside- or polymyxin-based treatment for MDR *P. aeruginosa* infections, especially in patients who may be at higher risk for aminoglycoside or nephrotoxicity, including those who are older and those with underlying comorbidities and illness [3,15]. Moreover, early treatment with ceftolozane/tazobactam may be prudent once susceptibly is known, as previous work has found that starting ceftolozane/tazobactam less than four days after the positive culture is associated with higher clinical and microbiological cure rates [12].

The main limitation to this work is that we were unable to distinguish between actual MDR *P. aeruginosa* infection and colonization. Only 23.6% of patients had a primary diagnosis of septicemia during admission, and the percentage with primary diagnoses of other infections assessed was only ~<5%. However, when diagnoses were considered anytime during admission, 82% had a diagnosis of an unspecified bacterial infection, 65% had septicemia, and 55% had pneumonia. However, all patients were treated with at least 48 h of ceftolozane/tazobactam, aminoglycosides, or polymyxins, and all MDR *P. aeruginosa* isolates were susceptible to the treatment of interest. 

Another limitation is that we did not assess the dosages or blood levels of study medications and cannot determine whether study drugs were appropriately dosed. Dosing strategies for aminoglycosides and polymyxins vary and optimal dosing targets are debated, resulting in potential overdosing or underdosing with aminoglycosides and polymyxins, as opposed to ceftolozane/tazobactam, which is generally dosed at 1.5 or 3 g every 8 h with normal renal function [7,16]. We also did not capture alternative routes of administration of the study drugs, such as via nebulization. 

We used a broad definition of previous and concomitant concurrent antibiotic treatments to control for all antibiotics used in addition to the treatment of interest. However, since we assessed all antibiotic exposures, it is possible that some of the antibiotic exposures were not solely for the treatment of the MDR *P. aeruginosa* infection. Ceftazidime/avibactam is another novel cephalosporin-beta-lactamase inhibitor therapy used for MDR *P. aeruginosa* infections that was approved in 2018; however, we did not include patients treated with ceftazidime/avibactam in our study. Due to their approvals after our study time frame, we also did not evaluate other more recently approved antibiotics, such as plazomicin, cefiderocol, and imipenem-cilastatin-relebactam [2]. 

Channeling bias may have influenced our study findings, as rates of acute kidney injury did not vary between the treatment groups. Patients at higher risk for acute kidney injury may have been preferentially treated with ceftolozane/tazobactam. The generalizability of this study may be limited to older male patients as this study was conducted among patients admitted to VA hospitals. Another limitation is that we assessed all-cause mortality and cannot rule out the impact of underlying conditions on mortality in our older, more clinically complex study population. 

## 4. Materials and Methods

This comparative effectiveness analysis included hospitalized VA patients with positive *P. aeruginosa* cultures between January 2015 and April 2018. Figure 1 presents a flow chart for the study of cohort identification. Multi-drug resistance was defined as any isolate that tested either intermediate (I) or resistant (R) to at least one antibiotic in at least three of these categories: extended-spectrum cephalosporins (cefepime, ceftazidime), fluoroquinolones (ciprofloxacin, levofloxacin), aminoglycosides (amikacin, gentamicin, tobramycin), carbapenems (imipenem, meropenem, doripenem), and piperacillin (piperacillin, piperacillin/tazobactam). We then included patients (1) treated with ceftolozane/tazobactam or aminoglycosides or polymyxins for ≥48 h without overlapping ceftolozane/tazobactam and aminoglycoside or polymyxin therapy for >48 h, (2) with MDR *P. aeruginosa* isolates susceptible to the treatment of interest (ceftolozane/tazobactam; or aminoglycoside or polymyxin-based regimens), and (3) treatment initiated one day prior through 5 days after culture collection. The index date was defined as the start date of the treatment of interest. 

We assessed the following objective clinical outcomes: inpatient mortality, 30-day readmission from discharge, persistent positive culture (defined as at least one subsequent positive culture after starting the treatment of interest, with and having received at least seven days of any antibiotic therapy), microbiological clearance (defined as a negative follow-up culture among those with follow-up cultures), and acute kidney injury (defined as a serum creatinine increase of 1.5 times the baseline serum creatinine).

We assessed several covariates, including demographics, clinical characteristics, current medical problems, medical history, infection diagnoses, culture source, resistance of the MDR *P. aeruginosa* isolate, and concomitant antibiotic treatment (Table 1). All antibiotic therapies received from the admission date until the index date were also evaluated. Concomitant treatments were defined as any antibiotic received from the index date through 15 days after initiation of the treatment of interest.

Baseline characteristics of patients in the ceftolozane/tazobactam and aminoglycoside- or polymyxin-based treatment groups were compared using the chi-square, Fisher’s exact test, *t*-test, or non-parametric Wilcoxon test, as appropriate. Confounding was assessed by controlling for variables significantly associated with the treatment of interest and the clinical outcome. Variables were included in the initial model if the univariate likelihood ratio *p*-value was ≤0.10 and were retained in the final model if the *p*-value was <0.05. aOR and 95% confidence intervals (CI) were calculated using automatic stepwise logistic regression. Different models were developed for each clinical outcome, each controlling for identified confounders of the specific exposure–outcome relationship.

## 5. Conclusions

In hospitalized patients with MDR *P. aeruginosa*, the risk of inpatient mortality was 61% lower among patients treated with ceftolozane/tazobactam compared with those treated with aminoglycoside or polymyxin-based regimens. Readmission, persistent positive cultures, microbiological clearance, and acute kidney injury did not differ between the treatment groups. Ceftolozane/tazobactam may be a treatment alternative to aminoglycosides or polymyxins for MDR *P. aeruginosa* infections, as it may have a more favorable safety profile and may be associated with improved clinical outcomes.

## Figures and Tables

**Table 1:** Baseline demographics and clinical characteristics of hospitalized patients with positive MDR *Pseudomonas aeruginosa* infections with ceftolozane/tazobactam or aminoglycoside or polymyxin treatment regimens.

Baseline Demographics and Clinical Characteristics	Ceftolozane/Tazobactam (n = 57)	Aminoglycosides/Polymyxins (n = 155)	*p*-Value
Age, years			
Mean (standard deviation)	67.5 (9.5)	71.1 (12.6)	0.03
Body mass index			
Mean (standard deviation)	27.4 (7.0)	27.1 (7.3)	0.77
Male	55 (96.5%)	154 (99.4%)	0.18
White	38 (66.7%)	109 (70.3%)	0.61
Hispanic or Latino	<5 (<8.8%)	35 (22.6%)	0.0003
Married	33 (57.9%)	63 (40.7%)	0.03
Admission source			
Home/community	24 (42.1%)	54 (34.8%)	0.33
Hospital	<5 (<8.8%)	12 (7.7%)	0.76
Nursing home	7 (12.3%)	5 (3.2%)	0.02
Treating specialty			
Medicine	19 (33.3%)	66 (42.6%)	0.22
Intensive care	14 (24.6%)	43 (27.7%)	0.64
Surgery	11 (19.3%)	12 (7.7%)	0.02
Other	12 (21.1%)	19 (12.3%)	0.11
Intensive care during admission	36 (63.2%)	89 (57.4%)	0.45
Surgery during admission	21 (36.8%)	57 (36.8%)	0.99
Charlson score			
Median (interquartile range)	5 (3–7)	3 (2–6)	0.01
Elixhauser score			
Median (interquartile range)	7 (4–10)	5 (3–8)	0.006
APACHE score			
Median (interquartile range)	40 (29–52)	43 (33–57)	0.29
Primary diagnosis			
Osteomyelitis	<5 (<8.8%)	<5 (<3.2%)	0.38
Pneumonia	<5 (<8.8%)	5 (3.2%)	0.44
Septicemia	16 (28.1%)	34 (21.9%)	0.35
Urinary tract infection	<5 (<8.8%)	12 (7.7%)	0.76
Other infections	<5 (<8.8%)	6 (3.9%)	0.19
Infection diagnosis during admission			
Bacterial Infection	50 (87.7%)	123 (79.4%)	0.16
Chronic osteomyelitis	12 (21.1%)	13 (8.4%)	0.01
Intraabdominal infection	7 (12.3%)	22 (14.2%)	0.72
Osteomyelitis	18 (31.6%)	28 (18.1%)	0.03
Pneumonia	30 (52.6%)	87 (56.1%)	0.65
Septicemia	40 (70.2%)	98 (63.2%)	0.35
Skin and Subcutaneous	13 (22.8%)	26 (16.8%)	0.31
infection			
Ulcers	23 (40.4%)	72 (46.5%)	0.43
Urinary tract infection	33 (57.9%)	97 (62.6%)	0.53
Current medical problems			
Respiratory failure	34 (59.7%)	89 (57.4%)	0.77
Fever of unknown origin	14 (24.6%)	30 (19.4%)	0.41
Acute renal failure	32 (56.1%)	88 (56.8%)	0.93
Shock	26 (45.6%)	55 (35.5%)	0.18
Complications of surgical	25 (43.9%)	55 (35.5%)	0.26
procedures or medical care			
Osteomyelitis	18 (31.6%)	30 (19.4%)	0.06
Spinal cord injury	22 (38.6%)	34 (21.9%)	0.02
Medical history			
Acute myocardial infarction	<5 (<8.8%)	10 (6.5%)	0.52
Congestive heart failure	21 (36.8%)	38 (24.5%)	0.08
Acute cerebrovascular disease	10 (17.5%)	19 (12.3%)	0.32
Cognitive disorders	14 (24.6%)	43 (27.7%)	0.64
Chronic obstructive	25 (43.9%)	60 (38.7%)	0.49
pulmonary disease			
Diabetes without	27 (47.4%)	75 (48.4%)	0.89
complications	22 (38.6%)	47 (30.3%)	0.25
Diabetes with complications	11 (19.3%)	41 (26.5%)	0.28
Malignancy	16 (28.1%)	47 (30.3%)	0.75
Metastatic solid tumor	8 (14.0%)	19 (12.3%)	0.73
Spinal cord injury			
Healthcare exposures, 30 daysprior to admission			
Hospitalization	17 (29.8%)	36 (23.2%)	0.33
Nursing home	<5 (<8.8%)	<5 (<3.2%)	0.02
Intensive care	<5 (<8.8%)	6 (3.9%)	0.70
Surgery	<5 (<8.8%)	9 (5.8%)	1.00
Length of hospital stay, days			
Median (interquartile range)	43 (16–80)	31 (13–107)	0.69
MDR *P. aeruginosa* culture source			
Respiratory	21 (36.8%)	55 (35.5%)	0.85
Urine	13 (22.8%)	80 (51.6%)	0.0002
Skin and tissue	10 (17.5%)	13 (8.4%)	0.06
Blood	8 (14.0%)	14 (9.0%)	0.29
Bone joint	7 (12.3%)	<5 (<3.2%)	<0.0001
Intra-abdominal	<5 (<8.8%)	<5 (<3.2%)	1.00
Other	5 (8.8%)	<5 (<3.2%)	0.03
Previous positive *P. aeruginosa* culture, prior 30 days	31 (54.4%)	60 (38.7%)	0.04
Culture source of prior positive *P. aeruginosa*			
Respiratory	14 (24.6%)	35 (22.6%)	0.76
Urine	10 (17.5%)	15 (9.7%)	0.12
Skin and tissue	5 (8.8%)	6 (3.9%)	0.17
Blood	6 (10.5%)	7 (4.5%)	0.11
Bone joint	<5 (<8.8%)	<5 (<3.2%)	0.06
Intra-abdominal	<5 (<8.8%)	<5 (<3.2%)	1.00
Other	<5 (<8.8%)	<5 (<3.2%)	0.06
Resistance			
Aminoglycosides	35 (61.4%)	82 (52.9%)	0.27
Carbapenem	53 (98.2%)	138 (91.4%)	0.12
Extended-spectrum	55 (96.5%)	138 (89.0%)	0.09
cephalosporin			
Fluoroquinolone	52 (91.2%)	142 (91.6%)	1.00
Piperacillin/tazobactam	35 (70.0%)	120 (88.2%)	0.003

**Table 2:** Treatment characteristics of hospitalized patients with positive MDR *Pseudomonas aeruginosa* infections with ceftolozane/tazobactam or aminoglycoside or polymyxin treatment regimens.

Treatment Characteristics	Ceftolozane/Tazobactam (n = 57)	Aminoglycosides/ Polymyxins (n = 155)	*p*-Value
Total number of changes in therapy during hospital admission			
Median (interquartile range)	7 (3–14)	6 (3–15)	0.92
Time to study drug from initial antibiotics during hospital admission, days			
Median (interquartile range)	13 (4–46)	9 (3–36)	0.27
Time to study drug from culture collection, days			
Median (interquartile range)	3 (1–4)	3 (2–4)	0.93
Inpatient antimicrobial duration, days			
Median (interquartile range)	34 (16–60)	23 (10–63)	0.18
Duration of study drug, days			
Median (interquartile range)	12 (5–18)	7 (4–14)	0.005
Number of changes in therapy before the start of study drug			
Median (interquartile range)	5 (1–10)	3 (1–8)	0.13
Number of days from hospital admission to start of study drug			
Median (interquartile range)	14 (4–48)	11 (4–46)	0.36
Time to antipseudomonal antibiotics * from admission, days			
Median (interquartile range)	0 (−2)	1 (0–6)	0.03
Any antibiotics, 30 days prior to the start of study drug	55 (96.5%)	144 (92.9%)	0.33
Previous antibiotics, 30 days to 8 days prior to the start of study drug			
Amikacin	<5 (<8.8%)	5 (3.2%)	1.00
Aztreonam	<5 (<8.8%)	<5 (<3.2%)	0.47
Cefepime	8 (14.0%)	20 (12.9%)	0.83
Ceftazidime	<5 (<8.8%)	<5 (<8.8%)	0.18
Cilastatin/imipenem	<5 (<8.8%)	8 (5.2%)	0.45
Ciprofloxacin	10 (17.5%)	15 (9.7%)	0.11
Daptomycin	9 (15.8%)	8 (5.2%)	0.02
Levofloxacin	9 (15.8%)	12 (7.7%)	0.08
Gentamicin	6 (10.5%)	5 (3.2%)	0.07
Meropenem	14 (24.6%)	18 (11.6%)	0.02
Piperacillin/tazobactam	20 (35.1%)	26 (16.8%)	0.004
Polymyxin B	<5 (<8.8%)	<5 (<3.2%)	0.57
Sulfamethoxazole/trimethoprim	7 (12.3%)	9 (5.8%)	0.14
Tobramycin	<5 (<8.8%)	<5 (<3.2%)	0.35
Vancomycin	24 (42.1%)	53 (34.2%)	0.29
Previous antibiotics class, 30 days to 8 days prior to the start of study drug			
Aminoglycosides	9 (15.8%)	13 (8.4%)	0.12
Carbapenem	15 (26.3%)	25 (16.1%)	0.09
Extended-spectrum	9 (15.8%)	21 (13.6%)	0.68
cephalosporin			
Fluoroquinolone	17 (29.8%)	25 (16.1%)	0.03
Piperacillin/tazobactam	20 (35.1%)	26 (16.8%)	0.004
Previous antibiotics, 7 days to 1 day prior to the start of study drug			
Amikacin	6 (10.5%)	<5 (<3.2%)	0.01
Aztreonam	<5 (<8.8%)	<5 (<3.2%)	0.29
Cefepime	9 (15.8%)	24 (15.5%)	0.96
Ceftazidime	6 (10.5%)	<5 (<3.2%)	0.03
Cilastatin/imipenem	<5 (<8.8%)	12 (7.7%)	0.36
Ciprofloxacin	5 (8.8%)	12 (7.7%)	0.78
Colistin	<5 (<8.8%)	<5 (<3.2%)	0.06
Daptomycin	7 (12.3%)	8 (5.2%)	0.13
Levofloxacin	5 (8.8%)	19 (12.3%)	0.48
Gentamicin	<5 (<8.8%)	<5 (<3.2%)	0.21
Meropenem	16 (28.1%)	26 (16.8%)	0.07
Piperacillin/tazobactam	19 (33.3%)	38 (24.5%)	0.20
Polymyxin B	<5 (<8.8%)	7 (4.5%)	0.68
Sulfamethoxazole/trimethoprim	<5 (<8.8%)	7 (4.5%)	0.73
Tobramycin	<5 (<8.8%)	<5 (<3.2%)	0.02
Vancomycin	27 (47.4%)	72 (46.5%)	0.91
Previous antibiotics class, 7 days to 1 day prior to the start of study drug			
Aminoglycosides	12 (21.1%)	8 (5.2%)	0.0004
Carbapenem	18 (31.6%)	37 (23.9%)	0.26
Extended-spectrum	15 (26.3%)	28 (18.1%)	0.18
cephalosporin			
Fluoroquinolone	10 (17.5%)	30 (19.4%)	0.75
Piperacillin/tazobactam	19 (33.3%)	38 (24.5%)	0.20
Concomitant antibiotics from start of study drug up to 15 days			
Aztreonam	<5 (<8.8%)	5 (3.2%)	1.00
Cefepime	9 (15.8%)	41 (26.5%)	0.10
Ceftazidime	<5 (<8.8%)	10 (6.5%)	1.00
Imipenem	<5 (<8.8%)	17 (11.0%)	0.09
Ciprofloxacin	<5 (<8.8%)	13 (8.4%)	0.36
Daptomycin	6 (10.5%)	13 (8.4%)	0.63
Levofloxacin	<5 (<8.8%)	18 (11.6%)	0.07
Meropenem	9 (15.8%)	47 (30.3%)	0.03
Piperacillin/tazobactam	14 (24.6%)	42 (27.1%)	0.71
Sulfamethoxazole/trimethoprim	<5 (<8.8%)	<5 (<3.2%)	0.39
Concomitant antibiotics class, start of study drug up to 15 days			
Carbapenem	11 (19.3%)	60 (38.7%)	0.008
Extended-spectrum	11 (19.3%)	47 (30.3%)	0.11
cephalosporin			
Fluoroquinolone	<5 (<8.8%)	30 (19.4%)	0.03
Piperacillin/tazobactam	14 (24.6%)	42 (27.1%)	0.71

* Treatment with any one of the following antipseudomonal antibiotics: amikacin, aztreonam, cefepime, ceftazidime, ceftazidime/avibactam, ceftolozane/tazobactam, ciprofloxacin, colistin, doripenem, gentamicin, imipenem, levofloxacin, meropenem, polymyxin B, piperacillin/tazobactam, tobramycin.

**Table 3:** Comparative effectiveness of ceftolozane/tazobactam compared with aminoglycoside or polymyxin treatment regimens among hospitalized patients with positive MDR *Pseudomonas aeruginosa* infections.

Outcomes	No. of Events/No. of Patients (%) Ceftolozane/Aminoglycosides/ Tazobactam Polymyxins		Adjusted Odds Ratio ^1^ (95% Confidence Interval)
Inpatient mortality	9/57 (15.8%)	43/155 (27.7%)	0.39 (0.16–0.93) ^5^
Readmission within 30 days of discharge	12/48 (25.0%)	31/112 (27.7%)	0.87 (0.40–1.89) ^6^
Persistent positive culture ^2^	7/31 (22.6%)	39/93 (41.9%)	0.38 (0.13–1.06) ^7^
Microbiological clearance ^3^	13/42 (31.0%)	33/108 (30.6%)	0.88 (0.35–2.21) ^8^
Acute kidney injury ^4^	8/48 (16.7%)	16/129 (12.4%)	0.86 (0.32–2.33) ^9^

^1^ Stepwise logistic regression. ^2^ Positive culture of index infection organism after 7 days of treatment. Denominator only includes patients with follow-up cultures. ^3^ Negative culture results at the site of index infection post index treatment administration. Denominator only includes patients with follow-up cultures. ^4^ Acute kidney injury only includes patients with baseline and follow-up serum creatinine. ^5^ Adjusted for prior positive *P. aeruginosa* culture, surgical treating specialty, and meropenem administered 30 days to 8 days prior to the start of index treatment. ^6^ No additional variables in the adjusted model. ^7^ Adjusted for prior positive *P. aeruginosa* culture. ^8^ Adjusted for *P. aeruginosa* culture site urine, marital status, and history of inflammation. ^9^ Adjusted for *P. aeruginosa* culture site urine and Charlson score.

## Data Availability

The study data may be made available upon reasonable request and approval by the Department of Veterans Affairs.
